# Booster Injection with Birch Pollen Extract Activates B‐Cellular Memory Responses in Patients Having Completed Allergen Immunotherapy

**DOI:** 10.1002/eji.70034

**Published:** 2025-08-17

**Authors:** Carolin Baum, Christian Möbs, Wolfgang Pfützner

**Affiliations:** ^1^ Clinical & Experimental Allergology Department of Dermatology and Allergology Philipps‐Universität Marburg Marburg Germany

**Keywords:** booster injections, allergen immunotherapy, birch pollen allergy, blocking IgG antibodies, memory B cells

## Abstract

Allergen immunotherapy (AIT) of patients with IgE‐mediated allergy results in the synthesis of blocking IgG antibodies mediating allergen tolerance. However, as antibody concentrations wane after stopping AIT, tolerance may be lost. The impact of a single booster allergen application on B‐cellular memory in AIT‐treated birch pollen (BP)‐allergic patients was investigated. Twenty‐five patients with BP allergy who finished AIT 3–12 years ago received one allergen injection approximately 4 months prior to the next BP season. We determined peripheral Bet v 1‐specific IgG‐secreting cells (ASC) by ELISPOT analysis and BP‐specific IgG, IgG4, and IgE serum antibodies by ImmunoCAP, and the allergen‐blocking capacity of IgG/IgG4 antibodies by ELIFAB assay. Clinical responses were assessed by visual analog scales. Immunological findings were compared with the primary B cell response of 12 BP‐allergic patients receiving conventional AIT. Bet v 1‐specific ASC significantly increased 2–4 weeks after BP‐injection, accompanied by enhanced levels of both BP‐specific IgG/IgG4 antibodies and allergen‐blocking serum activity. Compared with conventional up‐dosing, a single booster vaccination led to a markedly stronger B cell response after 4 weeks. Allergen booster injection activates B‐cellular memory associated with blocking IgG/IgG4 antibodies, pointing to reinforcement of allergen tolerance after completion of AIT.

## Graphical abstract

A single booster injection of allergen extract is capable of rapidly activating B‐cellular memory several years after completion of allergen immunotherapy, demonstrated by an increase of allergen‐specific IgG‐producing cells and IgG/IgG4 antibodies as well as improved allergen‐blocking capacity. This indicates that booster injections allow maintenance of long‐term allergen tolerance.

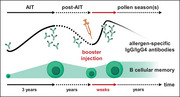

**Abbreviations**
AIT: allergen immunotherapy
ARC: allergic rhinoconjunctivitis
ASC: antibody‐secreting cells
BP: birch pollen
BPE: birch pollen extract
BPS: birch pollen season
ELIFAB: enzyme‐linked immunosorbent facilitated antigen binding
MBC: memory B cell
VAS: visual analog scale

## 1 Introduction

IgE‐mediated allergies have become one of the most serious challenges for public health. It is estimated that about 25–35% of the European population is affected by allergic rhinoconjunctivitis (ARC), putting respiratory allergies among the most common forms of chronic inflammatory diseases [1]. As avoidance of aeroallergens is not possible, allergen immunotherapy (AIT) with the aim of inducing allergen tolerance is the only causal therapy. A multitude of studies have shown clinical efficiency in patients with ARC receiving 3 years of AIT [2]. However, although persistence of treatment effects has been reported for 2–3 years after stopping AIT, long‐term follow‐up suggests progressive loss of tolerance in a considerable fraction of AIT‐treated patients over the following years [3, 4].

The establishment of allergen tolerance upon AIT is accompanied by a variety of immunological changes, which, among others, include the increased expression of immune regulatory cytokines like IL‐10, IL‐35, and TGF‐β, and the decreased secretion of allergy‐promoting type 2 cytokines like IL‐4, IL‐5, and IL‐13 [5]. For example, prominent T‐cellular effects comprise the induction of IL‐10‐producing type‐1‐regulatory T (Tr1) cells and loss of allergen‐specific Th2 cell activity [6, 7, 8]. Likewise, IL‐10‐secreting circulating follicular Th (Tfh) cells and innate lymphoid cells (ILC) are activated, while IL‐4‐producing Tfh cells, which promote IgE‐production by B cells, as well as IL‐5‐ and IL‐13‐secreting ILC are suppressed [9, 10]. The major B cell impact is the production of allergen‐blocking IgG antibodies, mainly of the IgG4 isotype, but also IgA antibodies [6, 11, 12]. As these antibodies compete with IgE, bound on effector cells, for the fixation of allergen, they take a pivotal part in committing allergen tolerance by preventing allergen‐induced mast cell and basophil degranulation and thus the elicitation of clinical symptoms [10]. Both IgG and IgG4 antibody concentrations and their allergen‐blocking activity, which can easily be assessed by IgE‐facilitated allergen binding assay [13, 14], continue to rise during the 3 years of repeated administration of AIT [15]. Besides, B cells have also been reported to contribute to the development of allergen tolerance by immunoregulatory activities, as they produce IL‐10 during AIT as well [16, 17, 18]. Their broad versatility as immune modulatory cells shaping the process of allergen tolerance is further underlined by supporting the induction of regulatory T (Treg) cells [10, 19].

While the immune mechanisms of AIT‐mediated allergen tolerance have been increasingly studied recently, less is known about the conditions ensuring its maintenance after AIT has been terminated. As the synthesis of allergen‐blocking antibodies during AIT is a crucial element of achieving allergen tolerance, an important question is how B‐cell memory can be preserved. It has been noticed that both the concentration and functional activity of allergen‐blocking IgG antibodies decline after finishing AIT [3, 15]. Thus, boosting allergen‐specific antibody production could present a promising option to sustain allergen tolerance. Booster vaccinations are a basic principle to achieve long‐term protection against viruses, microbial pathogens, or their toxins, consolidating a stable B cell response upon antigen encounter due to the activation of antigen‐specific memory BC (MBC) [20]. We thus wondered if the administration of birch pollen extract (BPE) as a booster vaccination in patients who were successfully treated with birch pollen (BP)‐AIT several years ago, might result in the stimulation of an allergen‐specific B‐cellular memory response with the aim to achieve long‐term increased concentrations of allergen‐blocking IgG antibodies. Recent studies have shown that sustained IgE responses share clonal origin with IgG memory and are most likely preserved by highly mutated, affinity matured IgG (particularly IgG1 and IgG4) MBC, which upon allergen challenge give rise to short‐living, IgE switched plasma blasts rather than by IgE MBC or by long‐living IgE secreting plasma cells [21, 22]. In this context, one could reason that the preformed allergen‐specific IgG MBC pool, shaped by a former AIT, can be reactivated by a single allergen injection to maintain an allergy protective IgG response.

## 2 Results

### 2.1 Birch Pollen Booster Injection Results in Increased Frequencies of Bet v 1‐specific IgG Antibody‐secreting Cells After 2–4 Weeks

**FIGURE 1:**
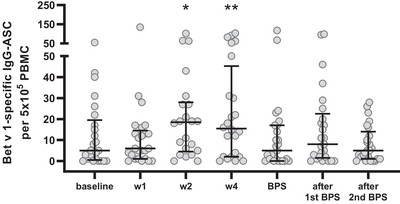
Alterations of Bet v 1‐specific IgG antibody‐secreting cells (ASC) after administration of a single booster dose of birch pollen extract. Frequencies of Bet v 1‐specific IgG‐ASC measured by ELISPOT assay increased until 4 weeks after booster injection, dropping to baseline values in the following birch pollen season (BPS). *p < 0.05 and **p < 0.01 as determined by using the Wilcoxon matched‐pairs signed‐rank test.

To determine if a booster injection with BP allergen leads to activation of allergen‐specific IgG antibody‐secreting cells (ASC), BP‐allergic patients treated by AIT 3–12 years ago with a relapse of symptoms of no more than 30% determined by visual analog scales (VAS) received a single injection of BPE (100,000 SQ‐U/ml) about 4 months prior to the next BP season (BPS). Peripheral blood samples were taken at different time points after booster vaccination. PBMC were stimulated with IL‐2 and the TLR agonist R848, and Bet v 1‐specific IgG‐ASC representing BP‐specific MBC were measured via ELISPOT assay (Figure 1; Figure **S1A**). A significant increase in ASC was noticed 2 and 4 weeks after booster injection, which had declined to baseline values upon the next BPS about 3 months later.

### 2.2 Allergen Booster Injection Leads to an Increase of Birch Pollen‐Specific IgG and IgG4 Antibodies, Correlating with the Induction of Bet v 1‐Specific Antibody‐Secreting Cells

**FIGURE 2:**
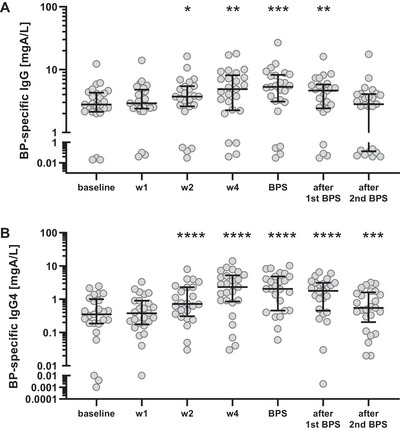
**Increase of birch pollen (BP)‐specific IgG and IgG4 antibodies in patients’ sera determined by ImmunoCAP analysis**. Serum concentrations of BP‐specific IgG (**A**) and IgG4 antibodies (**B**) increased after booster injection with BP extract as determined by ImmunoCAP analysis, giving rise to significantly higher values at the next and subsequent BP season (BPS) compared with baseline. *p < 0.05, **p < 0.01, ***p < 0.001, and ****p < 0.0001 as determined by using the Wilcoxon matched‐pairs signed‐rank test.

As allergen‐specific MBC differentiate into antibody‐producing plasmablasts upon stimulation, we wondered if BP‐specific IgG antibody concentrations would rise after a single booster injection with BPE. Analysis of serum from patients by ImmunoCAP who had received one booster vaccination with BPE showed a significant increase of BP‐specific IgG (Figure 2A; Figure S1B) and IgG4 antibodies (Figure 2B; Figure S1C) during the following weeks, which reached its peak around the subsequent BPS. Notably, Bet v 1‐specific IgG4 antibodies remained significantly elevated compared with the levels before injection during the complete observation time, which covered two BPS.

**FIGURE 3:**
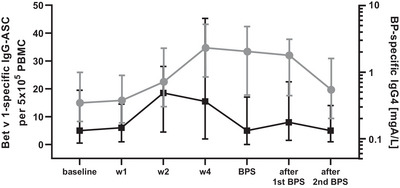
Interrelation between birch pollen (BP)‐specific IgG4 antibody concentrations and Bet v 1‐specific IgG antibody‐secreting cells (ASC). The number of allergen‐specific IgG‐ASC (■) reached its peak 2 weeks after injection, whereas IgG4 antibody levels (●) were highest at 4 weeks, but continued to stay elevated, with a decline becoming visible after the second BP season (BPS) after injection.

Enhanced frequencies of allergen‐specific ASC observed from week 2 to 4 after booster injection were closely followed by BP‐specific IgG4 antibody concentrations (Figure 3). While antibody levels peaked about 2 weeks after the maximum number of ASC, they stayed elevated for much longer time frames.

### 2.3 Allergen Booster Injection‐Induced Secretion of Birch Pollen‐Specific IgE Antibodies

**FIGURE 4:**
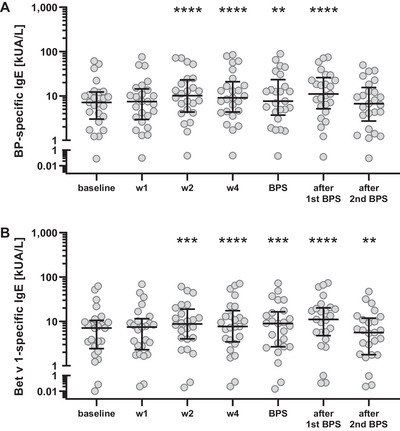
**Increased birch pollen (BP)‐specific IgE antibodies in patients’ sera after booster injection**. BP‐ (**A**) and Bet v 1‐specific IgE antibodies (**B**) increased upon booster injection, staying elevated until the end of the observation time, encompassing two BP seasons (BPS). *p < 0.05, **p < 0.01, ***p < 0.001, and ****p < 0.0001 as determined by using the Wilcoxon matched‐pairs signed‐rank test.

It has been noticed that allergen‐specific IgE antibodies show a temporary increase in the first weeks of AIT [6, 10, 15]. To examine the course of IgE‐synthesis after booster injection, we evaluated BP‐ as well as Bet v 1‐specific IgE in the patients’ sera. IgE concentrations significantly increased 1 and 2 weeks after allergen administration, respectively, showing enhanced levels also at further time points (Figure 4A,B; Figure S1D,E). Interestingly, at the end of the observation period, that is, after the second BPS, Bet v 1‐specific IgE concentrations decreased significantly compared with the baseline level (Figure 4B; Figure S1E).

### 2.4 Birch Pollen‐Specific IgG4 Antibody Response Dominates Compared with IgE Antibody Response

**FIGURE 5:**
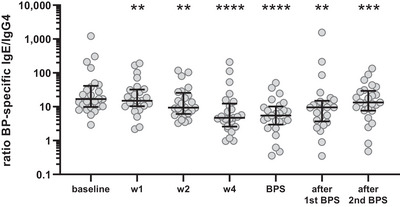
**Decreased ratio of birch pollen (BP)‐specific IgE/IgG4 upon allergen booster injection**. The ratio of BP‐specific IgE/IgG4 markedly decreased after booster injection with significantly lower values throughout the complete observation time covering two BP seasons (BPS). **p < 0.01, ***p < 0.001, and ****p < 0.0001 as determined by using the Wilcoxon matched‐pairs signed‐rank test.

To assess which antibodies predominate the immune response upon booster injection, we analyzed the proportion of allergen‐specific IgE compared with IgG4. The BP‐specific IgE/IgG4 ratio started to decline already one week after booster allergen vaccination and attained the lowest levels after 4 weeks until the next BPS (Figure 5; Figure S1F). Significantly decreased values were maintained through the complete observation time of two BPS. Thus, upon boosting, a higher amount of BP‐specific IgG4 antibodies was synthesized compared with IgE.

### 2.5 Birch Pollen Booster Injection Results in Increased Allergen‐Blocking Capacity of Patients’ sera

**FIGURE 6:**
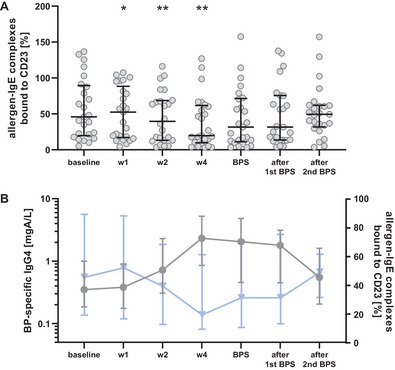
Bet v 1‐specific blocking capacity of sera from patients receiving a single booster injection of birch pollen (BP) extract. ELIFAB assay was performed to determine the blocking capacity of patients’ sera upon booster injection. (A) Blocking capacity increased significantly after BP vaccination, resulting in decreased formation of allergen‐IgE complexes. (B) Courses of BP‐specific IgG4 antibodies (●) and ELIFAB results (▼) showed that higher titers of BP‐specific IgG4 antibodies, as well as lower percentages of allergen‐IgE complexes bound to CD23, mirroring IgG BP blocking activity, coincided with the BP season (BPS) following booster vaccination. *p < 0.05, and **p < 0.01 as determined by using the Wilcoxon matched‐pairs signed‐rank test.

As the functional activity rather than serum concentrations of allergen‐specific IgG and IgG4 antibodies determines the clinical efficiency of tolerance induction, we investigated if the BP booster injection causes an increase in allergen‐blocking capacity, performing an enzyme‐linked immunosorbent facilitated antigen binding (ELIFAB) assay with the sera of BP‐vaccinated patients. An increased inhibitory potential of the sera upon booster injection was noticed, reaching its highest values after 4 weeks until the next BPS (Figure 6; Figure S1G). Thereafter, allergen‐blocking capacity gradually returned to baseline levels. Looking at both the course of BP‐specific IgG/IgG4 antibodies and the ability to form allergen‐IgE complexes, an inverse trend can be clearly observed (Figure 6B). The higher the concentration of BP‐specific IgG4‐antibodies, the higher the capacity to inhibit allergen‐binding by IgE antibodies, which started to diminish when BP‐specific IgG/IgG4 decreased.

### 2.6 Increased Numbers of Bet v 1‐Specific Antibody‐Secreting Cells Upon Booster Injection Are Accompanied by Fewer Clinical Symptoms During the Ensuing Birch Pollen Season

As BP booster injection results in elevated frequencies of allergen‐specific ASC associated with both increased concentration and enhanced blocking activity of BP‐specific IgG and IgG4 antibodies, we asked if individuals with higher numbers of Bet v 1‐specific ASC might show fewer clinical symptoms upon pollen exposure. Analysis of VAS data obtained during BPS demonstrated that during the first BPS, subjects with higher numbers of booster‐injection‐induced Bet v 1‐specific IgG‐ASC (≥50 spots) following allergen vaccination experienced fewer clinical symptoms than people with lower numbers (≤50 spots; **Figure** **S2**). However, these findings were not significant. Moreover, we could not find any correlation between the different parameters affected by the booster injection and the time interval, which had elapsed since the last application of an AIT dose (data not shown).

### 2.7 Booster Allergen Vaccination Results in a Rapid Re‐Activation of Allergen‐Specific B‐Cellular Memory Primed by Conventional, Primary Allergen Immunotherapy

**FIGURE 7:**
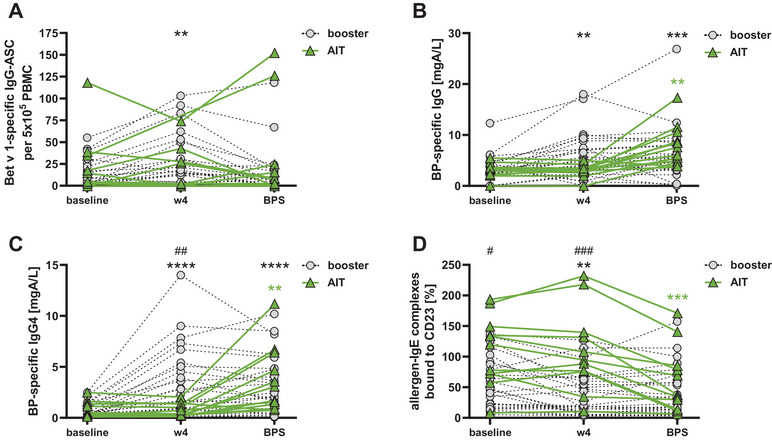
Comparison of primary B cell responses induced either by a single booster injection of birch pollen extract (BPE) or during the course of allergen immunotherapy (AIT) with BPE. Alterations of Bet v 1‐specific IgG‐ASC (A), concentrations of BP‐specific IgG (B) and IgG4 serum antibodies (C), as well as ELIFAB results (D) in patients who had received either a booster injection with BPE or conventional BPE‐AIT, respectively. **p < 0.01, and ****p < 0.0001 as determined by using the Wilcoxon matched‐pairs signed‐rank test. ^#^ *p* < 0.05, and ^##^ *p* < 0.01 as determined by using the nonparametric Mann–Whitney *U*‐test for unpaired samples.

To put the activation of B‐cellular memory upon a booster allergen vaccination in relation to the B‐cell response induced by primary AIT, we investigated another cohort of BP‐allergic individuals during the first months of conventional AIT. The observational period encompassed 12 weeks of administering weekly increasing doses of BPE and the following BPS within the maintenance phase of AIT, where the subjects got monthly BPE doses of 100,000 SQ‐U/mL (Figure 7; Figure S3). ELISPOT analyses of PBMC from AIT‐treated patients did not reveal a marked increase in Bet v 1‐specific ASC numbers after 4 weeks compared with the cohort, which had received a booster injection (Figure 7A). In addition, a delayed increase of allergen‐specific IgG and IgG4 antibodies, as well as their blocking activity, was noticed (Figures 7B–D). Of note, at this time point, AIT‐treated subjects had received a cumulative BPE dose of 4400 SQ‐U/mL, which was more than 20 times less than the booster dose. However, even after reaching the maintenance dose after 12 weeks (cumulative BPE dose of 320,400 SQ‐U/mL), concentrations of allergen‐specific IgG (3.87 [2.53–7.16] mgA/L) and IgG4 (0.9 [0.22–4.18] mgA/L) were still substantially lower compared with those of the booster cohort at week 4 (IgG: 4.89 [0.04–18] mgA/L, IgG4: 2.32 [0.03–14] mgA/L). Interestingly, BP‐allergic patients who had finished AIT 3–12 years ago showed a significantly higher allergen‐blocking serum activity at baseline than individuals treated with AIT for the first time (Figure 7D). Taken together, these data corroborate that a booster allergen vaccination of primarily AIT‐treated patients results in a swift reactivation of allergen‐specific memory B cells.

## 3 Discussion

AIT is a very effective measure for treating IgE‐mediated allergies like ARC against pollen by inducing allergen tolerance [2]. Therapeutic efficacy particularly rests on the increased production of allergen‐specific IgG antibodies, foremost of the IgG4 isotype, which upon natural exposure to the allergen prevents its binding by mast cell‐bound IgE antibodies and thus the elicitation of allergic symptoms [5, 15]. Three years of treatment are recommended to provide extended clinical benefit [23, 24]. However, successfully treated patients may lose AIT‐induced allergen tolerance over time after termination of AIT [3, 4]. As a major reason, the gradual loss of allergen‐blocking antibodies is considered [11, 15]. We here show that in BP‐allergic patients, treated by a 3‐year course of BP‐AIT 3–12 years ago, a single injection of BPE induces an allergen‐specific B‐cellular memory response, characterized by a rapid increase of Bet v 1‐specific ASC and the production of Bet v 1‐ as well as BP‐specific IgG and IgG4 antibodies. Despite receiving the full dose of maintenance AIT as a single shot, no serious side effects were observed, with most patients reporting only mild local injection reactions besides two experiencing a temporary, mild urticarial rash without systemic symptoms. It has been shown in patients with hymenoptera venom allergy that a modified AIT schedule with a single full‐dose injection of wasp venom without former up‐dosing was well tolerated [25]. Remarkably, significantly enhanced IgG4 antibody levels compared with preinjection concentration were observed during the complete observation period covering two consecutive BPS, which resulted in a persistent reduction of BP‐specific IgE/IgG4 ratio and were associated with a markedly increased allergen‐blocking capacity of patients’ sera in the first BPS, about 4 months after the booster vaccination.

B‐cellular memory rests on two pillars, one presented by long‐living plasma cells supplying a regular flow of affinity‐matured antibodies (constitutive humoral memory). The other consists of MBC, which are activated by antigen re‐exposure, resulting in the swift stimulation of a de novo, high‐magnitude production of antigen‐specific antibodies (reactive humoral memory) [26]. Thus, as the serum level of antibodies provided by continuous humoral memory mostly wanes over time, MBC take an important part in ensuring long‐term immunologic reactivity by replenishing the supply of newly formed, affinity‐matured antibody‐producing plasma cells [27]. Recent studies have shown that these cells not only circulate through the bloodstream, but also reside in epithelial tissues to quickly become activated by the encounter with foreign antigens [28]. Vaccination takes advantage of this pivotal immune principle, providing long‐term protection against infectious diseases by boosting pathogen‐specific immune responses [29, 30, 31, 32]. Our immunological results suggest that booster injections with allergens could similarly consolidate allergen tolerance in subjects formerly treated by AIT. Clinical efficiency seems to depend on the extent of the B‐cellular memory response, as individuals with a marked ASC increase (≥50 Bet v 1‐specific IgG‐ASC per 5 × 10^5^ PBMC) experienced stronger improvement of symptoms during the ensuing BPS compared with subjects with a lower rise of MBC. These findings are in line with vaccination data, considering vaccine responders to show at least >50 postvaccination spots of ASC detected by ELISPOT assay [33]. However, the results were not significant and have to be considered as preliminary, requiring placebo‐controlled trials with larger, well‐defined cohorts of allergic subjects. There has been another study examining the effect of a preseasonal intralymphatic booster injection in AIT‐treated grass pollen‐allergic patients, resulting in a significant increase of allergen‐specific IgG4 antibodies. Clinical symptom scores showed no difference between grass pollen extract and placebo‐treated subjects, and no further immunological parameters were evaluated, especially not the B‐cellular memory response [34].

Various studies investigating memory responses by ELISPOT‐analysis of PBMC in patients vaccinated with bacterial or viral antigens have demonstrated an increase of ASC, peaking after 2–4 weeks, which was followed by a differentially prolonged rise of antigen‐specific IgG antibodies [29, 30, 31, 35]. Re‐administration of allergen extract in AIT‐treated BP‐allergic patients showed a similar time course in the stimulation of ASC, but the serum concentration of allergen‐specific IgG antibodies and the blocking capacity seemed to decline more readily, although IgG4 levels were still significantly elevated at the end of the observation period covering two consecutive BPS. Humoral and MBC vaccine responses may be differently affected by various parameters like the nature of the antigen, age (with aged MBC being less able to differentiate in antibody‐secreting plasma cells) [35, 36], environmental influences or endogenous factors like the pre‐existing antibody titer, which has been reported to either promote or prevent MBC activation, by facilitating presentation of the applied antigen or blocking its uptake, respectively [31, 32]. However, no correlation could be identified between these different aspects and activation of ASC or antibody production in our cohort (data not shown). Obviously, natural allergen exposure during BPS had no additional booster effect.

BP booster injection resulted in both increased serum concentrations of BP‐specific IgG and IgE antibodies, which might imply that allergen‐specific IgE‐ASC become activated as well. These cells are thought to be mainly generated by sequential switching through IgG MBC and are barely detectable in humans [37]. A temporary rise of allergen‐specific IgE antibodies is a frequently observed effect in the induction phase of AIT [6, 10, 15]. However, both quantitatively and qualitatively, the impact of IgG‐ASC prevails, mirrored by the persistently decreased IgE/IgG4 ratio and the increased allergen‐binding capacity, respectively.

There are a few limitations of our study. First, the cohort is rather small and heterogeneous in terms of the time frame between finishing AIT and the booster allergen application (3–12 years). Furthermore, a placebo control is missing, thus hampering a definite statement regarding the clinical outcome. However, this trial presented a pilot study with the focus on immune parameters, in particular B‐cellular memory responses, in allergic subjects receiving a booster BP allergen injection. In order to retrieve meaningful long‐term results, it was conducted over a period covering two BPS. MBC activation involves affinity and avidity maturation of the secondarily produced antibodies. As these aspects may be of particular importance for bolstering allergen tolerance [38], it would be interesting to investigate them in more detail in prospective, more comprehensive clinical trials.

In summary, we show for the first time in a clinical pilot study, that a single booster injection of allergen extract in BP allergic individuals formerly treated by AIT results in a strong and—in comparison to patients treated with AIT the first time—rapid memory immune response, leading to activation and a significant rise of allergen‐specific ASC with a subsequent prolonged increase of allergen‐blocking IgG antibodies. This approach might hold promise in consolidating allergen tolerance in subjects with waning IgG antibody levels at risk of experiencing a relapse of allergy symptoms.

## 4 Materials and Methods

### 4.1 Study Subjects

**FIGURE 8:**
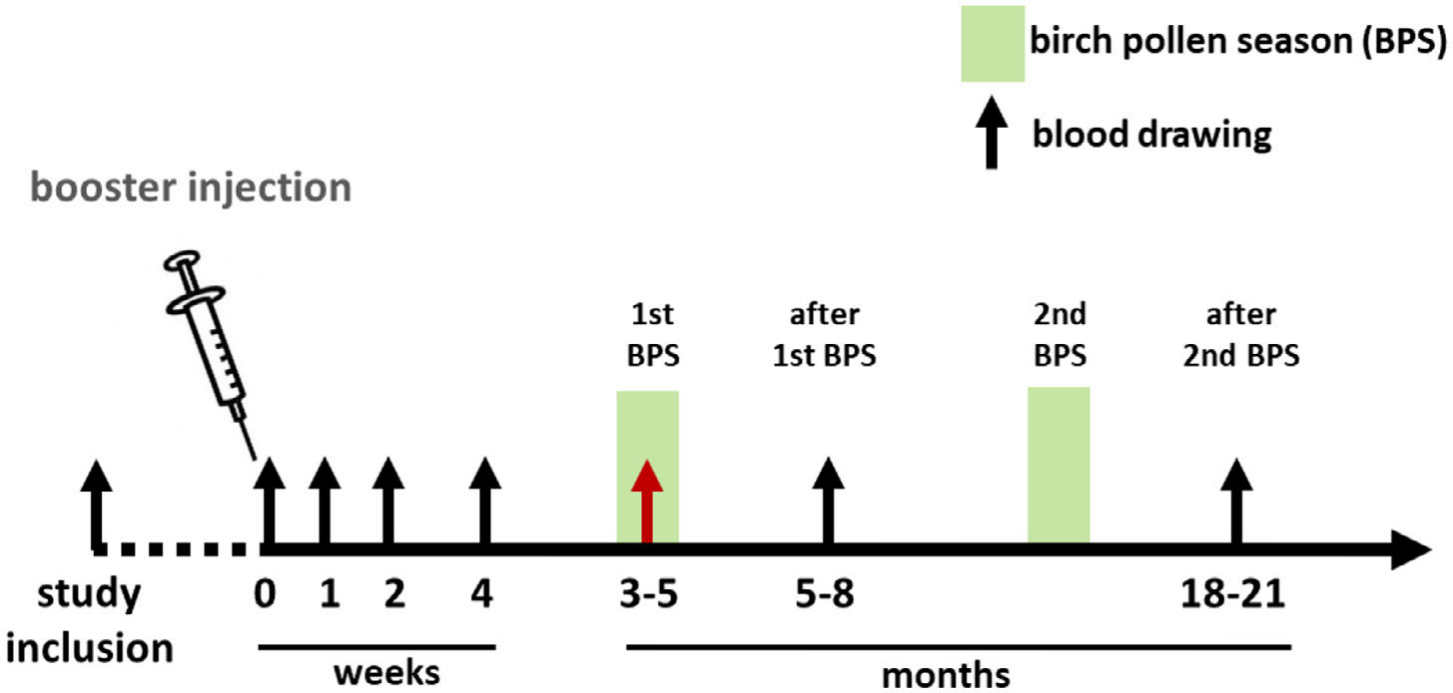
Clinical trial design. Birch pollen (BP)‐allergic patients treated with BP allergen immunotherapy 3–12 years ago received a single booster injection with BP extract, approximately 4 months prior to the next birch pollen season (BPS; week 0). Blood was drawn at several time points as indicated by arrows.

The study consisted of a booster vaccination cohort of 25 patients (age 36–63 years, 17 females) and another cohort of 12 subjects treated with conventional AIT (age 23–60 years, 5 females), both diagnosed with ARC to BP by clinical history, positive skin prick test and BP‐specific IgE serum antibodies >0.35 kUA/L. Patients receiving a BP booster injection had successfully completed AIT 3 to 12 years ago, with a relapse of symptoms of no more than 30%, compared with the clinical condition after completion of AIT as judged by VAS [39, 40, 41]. Exclusion criteria were symptomatic perennial respiratory allergies, allergic and nonallergic asthma, an ongoing AIT, severe respiratory, cardiovascular, or chronic inflammatory diseases, as well as immunosuppressive medication. Approximately 4 months prior to the next BPS, the subjects of the booster cohort received a single dose of 100,000 SQ‐U/mL (ALK‐depot SQ Birch; ALK‐Abelló, Hamburg, Germany), subcutaneously injected at the dorsal upper arm, about 10 cm proximal to the elbow. Patients were clinically monitored for 30 min regarding the occurrence of side effects. Clinical symptoms during the following two BPS were assessed shortly after the end of each season by VAS, with marks within 0–30 mm representing mild, within 31–70 mm moderate, and 71–100 mm severe symptoms. Blood was drawn and PBMC were cryopreserved at different time points, namely at the day of study inclusion, directly before administration of BPE (week 0), 1, 2, and 4 weeks after booster injection, during the next BPS (month 3–5), and shortly after this season (month 5–8) and the next thereafter (month 18–20; Figure 8). In accordance with the manufacturer's established 11‐injection up‐dosing schedule, patients of the AIT cohort received preseasonal, incremental, weekly doses of birch pollen allergen (up‐dosing phase; ALK‐depot SQ, ALK‐Abelló) administered subcutaneously until a dose of 100,000 standard quality‐units per injection was reached, which was given thereafter in monthly intervals (maintenance phase). For comparative analysis, blood was taken and PBMC cryopreserved at week 0, 4, and during the following BPS after initiation of AIT. The study was approved by the local Ethics Committee of the Medical Faculty of the Philipps‐Universität, Marburg, Germany (Az. 24‐13 and 25‐13). All patients had provided written informed consent to participate in the trial.

### 4.2 Blood Samples and Preparation of PBMC

Citrate‐phosphate‐dextrose‐adenine (CPDA)‐containing peripheral blood samples were taken from the patients at defined time points (Figure 8). PBMC were isolated by using density gradient centrifugation (Lymphocyte Separation medium, Capricorn Scientific GmbH, Ebsdorfergrund, Germany), and a total of 10^7^ PBMC per milliliter were frozen in 50% heat‐inactivated FCS (Biochrom GmbH, Berlin, Germany) and 10% dimethyl sulfoxide (Sigma, Schnelldorf, Germany). After initial gradual freezing (decrease by 1°C/min) in freezing containers (Nalgene Nunc International, Rochester, NY, USA) to −80°C, PBMC were kept in liquid nitrogen for long‐term storage.

### 4.3 Serum Immunoglobulins

Serum concentrations of total IgE, as well as specific IgE, IgG, and IgG4 antibodies against Betula verrucosa (birch) pollen allergen, and specific IgE antibodies against the major BP allergen Bet v 1 were measured by using the Phadia ImmunoCAP System, according to the manufacturer's instructions (Phadia/Thermo Fisher, Freiburg, Germany).

### 4.4 ELIFAB Assay

ELIFAB assay was performed according to the protocol described by Shamji et al. [13, 14]. In brief, indicator serum containing a high concentration of Bet v 1‐specific IgE (>100 kUA/L) was preincubated with rBet v 1 (Biomay, Vienna, Austria) at 37°C for 1 h to form allergen‐IgE complexes. To assess the inhibition of facilitated allergen binding, 20 µl of indicator serum was either mixed with 20 µL of test serum or an equal volume of medium as a control. After preincubation, allergen‐IgE complexes were transferred to soluble (s)CD23‐coated plates (R&D Systems, Wiesbaden‐Nordenstadt, Germany). By adding biotin‐conjugated anti‐human IgE antibodies (BD Biosciences, Heidelberg, Germany), streptavidin‐peroxidase, and 3,3’,5,5’‐tetramethylbenzidine (TMB) substrate (both Sigma‐Aldrich), bound allergen‐IgE complexes were detected. All samples were measured in duplicates. Data were expressed as binding of allergen‐IgE complexes relative to the binding with indicator serum alone.

### 4.5 Bet v 1‐Specific IgG ELISPOT Assay

An IgG ELISPOT assay was used to detect allergen‐specific IgG‐ASC representing circulating Bet v 1‐specific MBC [42, 43]. After thawing, PBMC were stimulated at 1 × 10^6^ cells/mL with 10 ng/mL recombinant human (rh)IL‐2 and 1 µg/mL TLR agonist R848 [44], or were left unstimulated and subsequently incubated for 6 days at 37°C in a humidified atmosphere containing 5% CO_2_. ELISPOT plates were prewetted with 70% ethanol for 2 min and washed with sterile water. For the detection of Bet v 1‐specific IgG‐ASC, anti‐IgG coating monoclonal antibodies were diluted to a concentration of 15 µg/mL in sterile PBS and added to the plates. Afterwards, the plates were incubated overnight at 4°C. Plates were washed with PBS and blocked with cell culture medium supplemented with 10% FCS for 1 h at room temperature (RT). After the prestimulation period, cells were harvested and added to the plate at concentrations of 5 × 10^5^ PBMC/well for the detection of allergen‐specific IgG‐ASC. Wells only incubated with medium were used as blank controls. ELISPOT plates were incubated for 20 h at 37°C + 5% CO_2_. Cells were discarded, and plates were washed with PBS. To detect Bet v 1‐specific IgG‐ASC, biotinylated Bet v 1 was utilized at a concentration of 1 µg/mL diluted in PBS + 0.5% FCS. Streptavidin‐horseradish peroxidase (SA‐HRP) diluted 1:100 in PBS + 0.5% FCS was added to the plates for 1 h at RT. Thereafter, 3‐amino‐9‐ethylcarbazole (AEC) substrate solution was applied for the color development of spots. Spots were counted using a plate reader, and mean numbers of Bet v 1‐specific spots were determined in duplicates. The number of spots of unstimulated samples was subtracted from the number of spots of the respective stimulated samples.

### 4.6 Statistical Analysis

Data were expressed as median with interquartile range. Comparison of paired samples before and at different time points after booster injection was done by using the nonparametric Wilcoxon signed‐rank test with GraphPad Prism software (version 9.1.0; GraphPad Software, La Jolla, CA, USA). Comparison of unpaired samples (booster cohort vs. AIT cohort) at the same time points was performed using the nonparametric Mann–Whitney *U*‐test. Differences were considered statistically significant at *p*‐values of less than 0.05.

## Author Contributions

Christian Möbs and Wolfgang Pfützner designed the study. Carolin Baum performed the experiments. Carolin Baum and Christian Möbs analyzed the data and performed the statistical analysis. All authors wrote and approved the manuscript for publication.

## Conflicts of Interest

Wolfgang Pfützner reports compensation for consulting/scientific advisory board membership and/or received research grants from ALK‐Abelló, Almirall, Janssen‐Cilag, Leo Pharma, Phadia/Thermo Fisher, and Sanofi. The remaining authors declare no conflicts of interest.

## Ethics Approval Statement for Human Studies

The study was approved by the local Ethics Committee of the Medical Faculty of the Philipps‐Universität Marburg, Marburg, Germany (Az. 24‐13 and 25/13).

## Patient Consent Statement

All patients had provided written informed consent to participate in the trial.

## Peer Review

The peer review history for this article is available at https://publons.com/publon/10.1002/eji.70034.

## Supporting information




**Supporting File 1**: eji70034‐sup‐0001‐SuppMat.pdf.

## Data Availability

The data that support the findings of this study are available from the corresponding author upon reasonable request.
