# Supplementary material for: Booster Injection with Birch Pollen Extract Activates B‐Cellular Memory Responses in Patients Having Completed Allergen Immunotherapy
Source: Eur J Immunol. 2025 Aug 17;55(8):e70034. doi: 10.1002/eji.70034 (PMC12358704; doi:10.1002/eji.70034)
Supplement: Supplementary file 1 — Supporting File 1: eji70034‐sup‐0001‐SuppMat.pdf. [file EJI-55-e70034-s001.pdf]

Supporting Information

Booster injection with birch pollen extract activates B cellular memory responses in patients having completed allergen immunotherapy

Carolin Baum, Christian Möbs and Wolfgang Pfützner

Clinical & Experimental Allergology, Department of Dermatology and Allergology, Philipps-Universität Marburg, Marburg, Germany

Supplemental Figures

Supplementary Figure S1. Individual courses of B cellular responses in patients after receiving a single booster injection of birch pollen (BP) extract.

Supplementary Figure S2. Increased numbers of allergen-specific antibody-secreting cells (ASC) after booster injection are associated with less clinical symptoms during natural allergen exposure.

Supplementary Figure S3. Comparison of allergen-specific IgE responses induced either by a single booster injection of birch pollen (BP) extract or during the course of allergen immunotherapy (AIT) with BP extract.

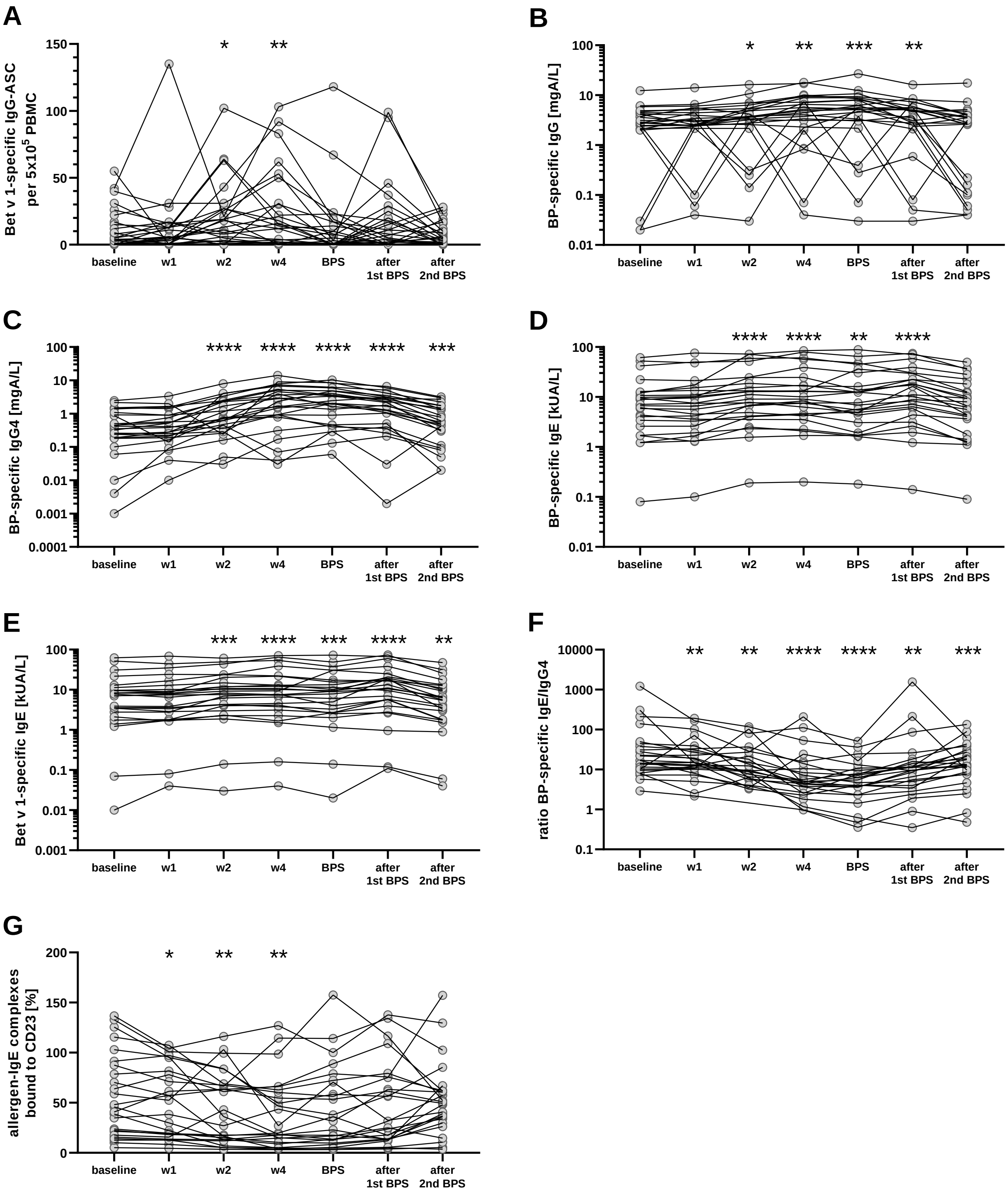

Supplementary Figure S1. Individual courses of B cellular responses in patients after receiving a single booster injection of birch pollen (BP) extract. (**A**) Frequencies of Bet v 1-specific IgG-ASC measured by ELISPOT assay. BP-specific IgG (**B**), IgG4 (**C**) and IgE (**D**) as well as Bet v 1-specific IgE antibodies (**E**) in patients' sera determined by ImmunoCAP analysis. (**F**) Ratio of BP-specific IgE/IgG4. (**G**) Bet v 1-specific serum blocking capacity analyzed by ELIFAB assay. *p < .05, **p < .01, ***p < .001, and ****p < .001 as calculated by using the Wilcoxon matched-pairs signed-rank test.

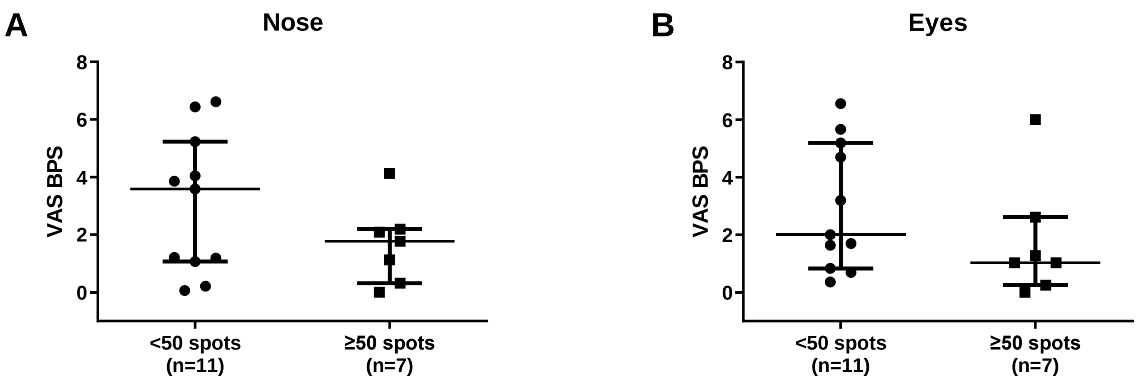

Supplementary Figure S2. Increased numbers of allergen-specific antibody-secreting cells (ASC) after booster injection are associated with less clinical symptoms during natural allergen exposure. Individuals with enhanced numbers of Bet v 1-specific ASC (> 50 spots, detected 2 to 4 weeks after allergen vaccination by ELISPOT) experienced less clinical nose (rhinorrhea, obstruction, sneezing, itch; **A**) and eye (watery eyes, redness, itch; **B**) symptoms during the first birch pollen season (BPS) after booster injection (recorded by visual analog scale (VAS)), compared to subjects with lower frequency of ASC.

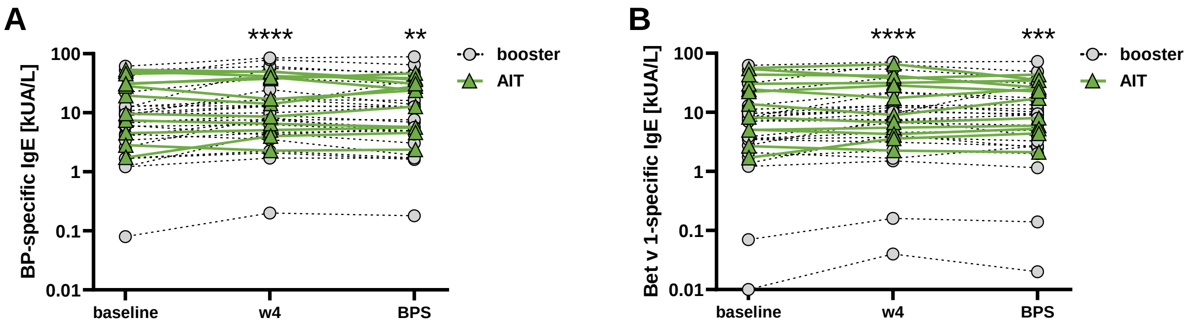

Supplementary Figure S3. Comparison of allergen-specific IgE responses induced either by a single booster injection of birch pollen (BP) extract or during the course of allergen immunotherapy (AIT) with BP extract. Alterations of concentrations of BP- (**A**) and Bet v 1-specific (**B**) IgE serum antibodies in patients receiving a booster injection with BP extract or BP-specific AIT, respectively. **p < .01, ***p < .001, and ****p < .0001 as determined by using the Wilcoxon matched-pairs signed-rank test.
